# Level of option B plus drug adherence for preventing mother-to-child transmission of HIV and associated factors among HIV-positive women in the awi zone, amhara region, northwest Ethiopia,2020

**DOI:** 10.1016/j.heliyon.2024.e35319

**Published:** 2024-07-26

**Authors:** Tegegne Wale Belachew, Besfat Berihun Erega, Mesafint Ewunetu, Kihinetu Gelaye, Tigist Seid Yimer, Wassie Yazie Ferede

**Affiliations:** a Department of Midwifery College of Medicine and Health Sciences, Debre Tabor University, Ethiopia; b Department of Midwifery College of Medicine and Health Sciences, Bahir Dar University, Ethiopia

**Keywords:** Adherence, Option B plus, PMTCT, Awi zone, ART, HIV/AIDS

## Abstract

**Introduction:**

Adherence to Option B+ antiretroviral medication (ART) is essential for the successful implementation of the Prevention of Mother-to-Child Transmission (PMTCT) program. However, poor adherence to Option B + PMTCT drugs among women results in increased viral load and mother-to-child transmission and reduces immunological and clinical outcomes.

**Objective:**

The objective of the study was to assess the level of Option B plus drug adherence for preventing mother-to-child transmission of HIV and associated factors among HIV positive women in selected government health facilities of Awi zone, Amhara region, Northwest Ethiopia,2020.

**Methods:**

This institutional-based cross-sectional study was conducted from March 1 to April 30 among 358 HIV-positive women (pregnant and lactating mothers). A multistage sampling procedure was used to select the study participants. Data were collected using a structured questionnaire through interviews. The collected data were entered into EPI Data 3.1 statistical software for data management and analyzed using SPSS version 25 statistical package. The associations between variables were analyzed using bivariate and multivariable logistic regression models. A p-value ≤0.05 at the 95 % confidence interval was considered statistically significant.

**Results:**

Out of the 358 participants, adherence to Option B + PMTCT was 83.24 %. The study revealed that counselling [AOR = 4.4, 95 % CI: 1.60–12.29], partner support involvement [AOR = 3.0, 95 % CI: 1.17–7.92], and time taken to reach from home to the facility [AOR = 3.1, 95 % CI: 1.51–6.52] were significantly associated with the level of adherence to Option B + PMTCT.

**Conclusion:**

This study showed that the level of Option B + PMTCT drug adherence was lower than the nationally recommended adherence level. Good counselling, partner support, and reduced travel time from home to the facility were associated with adherence to Option B + PMTCT drugs. Therefore, counselling is crucial for increasing adherence to Option B + PMTCT drugs. Accessible health facilities reduce travel burdens, encourage regular clinic visits, and enhance adherence to PMTCT drugs. Partners can provide reminders, attend appointments, offer emotional support, and explore alternatives such as mobile clinics or medication delivery services.

## 1 Introduction

Millions of children are affected by the global HIV/AIDS pandemic [1]. Mother-to-child transmission is the most common mode of acquiring HIV infection in children. It is estimated that without the use of antiretroviral therapy (ART), approximately 20–25 % of HIV transmission occurs during pregnancy, 30–50 % of HIV transmission occurs during labour and delivery, and 25–45 % of HIV transmission occurs during breastfeeding period [2]. The risk of HIV transmission can decrease to less than 5 % for breastfeeding women and less than 2 % for those who are not breastfeeding [3].

To eradicate new cases of pediatric HIV transmission, the World Health Organization (WHO) updated its guidelines for preventing mother-to-child transmission (PMTCT) of HIV in 2010. The guidelines proposed two options (Option A and Option B) focused on preventing transmission during pregnancy and breastfeeding. In option A, pregnant women start taking zidovudine at 14 weeks of pregnancy and continue the medication throughout their pregnancy. Infants receive nevirapine throughout the breastfeeding period [4].

In option B, pregnant women receive triple antiretroviral (ARV) prophylaxis starting at 14 weeks of pregnancy and continuing until the end of the breastfeeding period. Infants receive nevirapine during the first four to six weeks after birth. Both options recommend lifelong antiretroviral therapy (ART) for all women in WHO clinical stage three or four or with a CD4 cell count of 350 cells/mm3 or lower. The use of antiretroviral prophylaxis is expected to decrease the risk of HIV transmission to breastfeeding infants from 35 % to 5 % [4].

In Option B+, the World Health Organization (WHO) advises initiating lifelong triple antiretroviral therapy for all pregnant and breastfeeding women living with HIV, regardless of WHO clinical stage, gestational age, or CD4 cell count [5].

Adherence refers to the degree to which a patient's actions, such as medication intake, following a prescribed diet, or embracing healthy lifestyle modifications, align with the established guidance provided by a healthcare provider [6].

Optimal adherence to antiretroviral therapy (ARV) is crucial for preserving maternal health, minimizing the risk of medication resistance, and preventing HIV transmission to children [7].

Ethiopia has adopted WHO strategy aimed at achieving adherence levels of 95 % or higher [8]. Enhanced immunological and clinical results, reduced viral load, and decreased transmission risk are associated with higher adherence to ARV drugs. Conversely, inadequate adherence to Option B + PMTCT can lead to viral resistance, immunological deterioration, and an elevated risk of mother-to-child HIV transmission [9].

Ensuring adherence to Option B + ART medication is essential for all HIV-positive pregnant and breastfeeding women to enhance maternal health, prevent mother-to-child transmission of HIV, and reduce the viral load on sexual partners [10]. This approach may have played a role in reaching the goal of ending the HIV/AIDS epidemic by Ref. [11]. Option B+ stands out as a more effective PMTCT strategy due to its ability to overcome barriers and achieve broad treatment coverage [5].Streamlining drug regimen choices could enhance mothers' adherence and facilitate higher adherence rates [12].

Every year, approximately 1.4 million HIV-infected women globally give birth in regions where PMTCT services are restricted [13]. In 2009, more than 370,000 children contracted HIV through mother-to-child transmission [14].

Approximately 25 % of pregnant women experience suboptimal adherence to ART, with adherence rates worsening during the postpartum period. Even among women who remain in medical care, adhering to ART medication proves to be challenging. England faces significant obstacles that impact treatment adherence during pregnancy and the postpartum phase [15].

Numerous studies conducted in Africa indicate that the adherence rate fell below 95 %, signalling poor adherence levels. In Mpumalanga Province, South Africa, the adherence to ART drugs among HIV-positive pregnant women was recorded at 69 % [16].After one year postpartum, only 40 % of women in Zimbabwe were continuing to take ART [17].

A meta-analysis study conducted by Nachega et al. on adherence to ART in pregnancy and postpartum within PMTCT programs revealed that 73.5 % of pregnant women across low-, middle-, and high-income nations displayed suboptimal adherence. Additionally, adherence to ARVs was noted to be higher during pregnancy compared to the postpartum period [15].

Challenges of option B plus medication adherence include insufficient awareness, inadequate support and disclosure from partners, apprehension regarding prejudice and stigma, substandard clinical practices, deficiencies in provider training and knowledge, limited access to resources, and healthcare workers' attitudes [18,19].

Insufficient adherence to Option B + PMTCT ARV medication during lactation could lead to an increased risk of virus transmission from mother to child [9]. The meta-analysis study conducted by Kassa et al. in Ethiopia on the pooled prevalence of mother-to-child transmission (MTCT) of HIV was 9.93 % [20].

Ethiopia adopted a national policy aimed at achieving adherence to ART of 95 % or higher to enhance general health and reduce viral loads. However, a study conducted in the South Wollo Zone of Northeast Ethiopia found that 87.9 % of pregnant and lactating mothers adhered to Option B + PMTCT ART medication [21,22].

Studies on adherence to Option B + PMTCT medications in Ethiopia have not been conducted on mothers who breastfeed from 6 to 18 months. This study incorporates HIV-positive women who breastfeed from birth to 18 months. Additionally, no research has been conducted on adherence to Option B PMTCT medication in the Awi Zone. The results of this study have helped to obtain new knowledge on adherence to Option B + PMTCT drugs, as this program began in Ethiopia in 2012. This research can help improve the effectiveness of PMTCT programs in Ethiopia and contribute to supporting the elimination of MTCT in the study area and other parts of the country. It can also enable pregnant and lactating mothers to seek intervention, adhere to their treatment, and understand the challenges and facilitators that affect adherence to antiretroviral treatment among pregnant and breastfeeding women living with HIV.

Therefore, the aim of the study was to assess the level of adherence to Option B + PMTCT drugs and associated factors among HIV positive women in selected government health facilities in Awi Zone, Amhara Region, Northwest Ethiopia.

## 2 Methods and materials

### 2.1 Study design and period

An institutional-based cross-sectional study design was carried out from March1- April 30/2020 among 358 HIV positive women.

### 2.2 Study area

The research was conducted in specific government health facilities located within the Awi zone, one of the 13 zones in the Amhara region. Injibara, the capital of the Awi zone, is situated 445 km from Addis Ababa and 114 km from Bahir Dar, spanning an altitude range of 1800–3100 m above sea level. According to the 2017 population projection, the Awi zone has a total population of 1,196,196 individuals, comprising 602,925 females and 593,271 males. Within this zone, there are 47 health centers and five public hospitals, all of which offer services under Option B.

### 2.3 Source population

All HIV-positive women who underwent PMTCT follow-up in governmental health facilities in the Awi zone were included in the study.

### 2.4 Study population

Selected HIV-positive women who received PMTCT follow-up care at selected governmental health facilities in the Awi zone during the study period were included in the study.

### 2.5 Inclusion criteria

The study included pregnant and breastfeeding women who had received PMTCT follow-up for a minimum of one month at selected public health facilities in the Awi zone.

### 2.6 Exclusion criteria

Pregnant and breastfeeding women who were undergoing PMTCT follow-up but were critically ill (such as being unconscious and requiring emergency treatment) were excluded from the study.

### 2.7 Sample size determination

The sample size for the first objective was determined using the single-population proportion formula with a 95 % confidence interval (CI), a 5 % margin of error, and an estimated adherence proportion of 87.9 %, as obtained from a study conducted in southern Wollo, Amhara region, northwestern Ethiopia [23]. The formula used is n = Z^2^ p (1-p)/w^2^ = (1.96)^2^(0.879 × 0.121)/(0.05)^2^ = 163.

After considering a 10 % nonresponse rate, the overall sample size was adjusted to 179.

With a design effect of 2, the total sample size was doubled to 358.

The sample size for the second objective was determined using Epi Info version 7, considering the following assumptions: a 95 % confidence interval (CI), 80 % power, a 1:1 ratio, and P1 (prevalence of the exposed group) and P2 (prevalence of the unexposed group) derived from a previous study in the Hadia zone [24]((Table 1). Finally, the largest sample size of 358 was determined based on proportion.

**Table 1:** The calculated sample size for Objective Two involves using associated factor.

Tittle	Factors	Prevalence	Power	OR	The sample size includes a 10 % non-response rate and a design effect of two
Option B + PMTCT drug adherence and associated factors among women in the Awi zone, Amhara region, northwest Ethiopia	Counselled side effect of ART drug	P1 = 60.6 % P2 = 94.1 %	80 %	10.4	134
	Disclosure HIV status	P1 = 53.2 % P2 = 92.9 %	80 %	0.1	106

### 2.8 Sampling technique and procedure

**Fig. 1:**
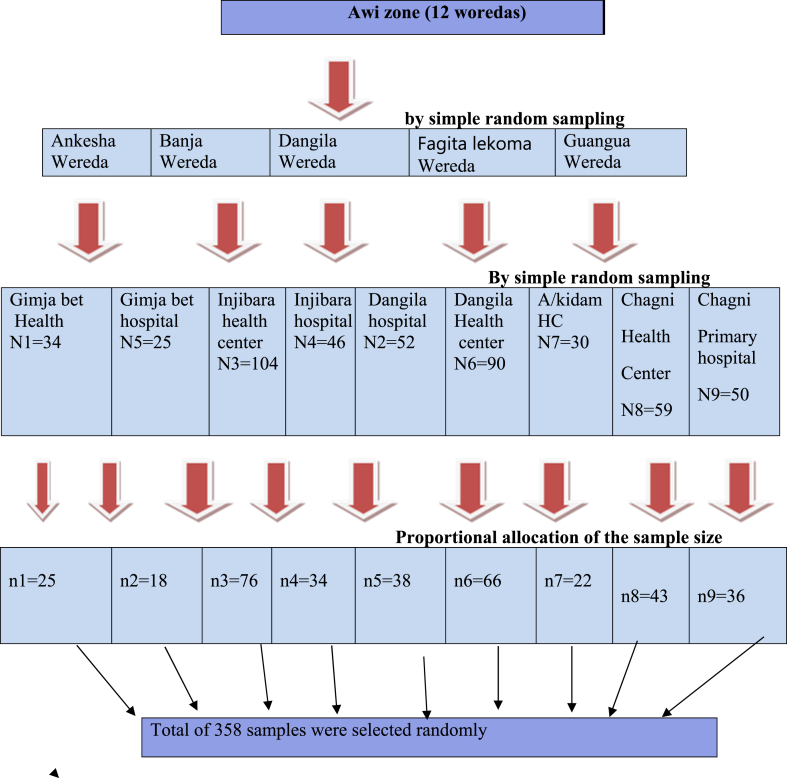
Schematic presentation of sampling procedure among the respondents in selected health facility of Awi zone, Northwest Ethiopia, March 1-April 30, 2020(N = 358.

A multistage sampling procedure was performed to select the study participants. In stage one, five out of the twelve woredas in the Awi zone were selected randomly. In stage two, health facilities within each woreda were also selected randomly. In stage three, samples were distributed proportionally among the selected health facilities as follows: Dangila Hospital [25], Gimjabet Hospital [18], Injibara Hospital [26], Chagni Primary Hospital [27], Injibara Health Center (76), Dangila Health Center (66), Addis Kidam Health Center [22], Gimjabet Health Center [28], and Chagni Health Center (43). The sampling frame, derived from the PMTCT cohort registration book, was utilized for this purpose. A random sampling technique using the lottery method was employed to select the study subjects. Participants were interviewed during their follow-up at the selected public health facilities (Fig. 1).

### 2.9 Operational definition of variables

Level of adherence – Mothers' adherence to Option B + PMTCT medicine was assessed using self-reporting to determine their adherence level compared to other mothers. Responses indicated either a good or low degree of adherence based on their self-reported adherence to the regimen. HIV-positive women who answered "No" to each of the four adherence questions were considered to have a good level of adherence. Participants who answered "Yes" to at least one of the four questions were classified as nonadherent [28].

Knowledge**:** The level of knowledge for the Option B + PMTCT program was assessed based on the total number of correct answers to eight knowledge questions. Women scoring ≥80 %, 60–79 %, and <60 % were categorized as having high, moderate, and low knowledge, respectively [29].

Male partner involvement - In terms of male partner support, involvement was categorized as good for those who scored ≥7, moderate for those who scored 4–6, and low for those who scored ≤3(26).

Disclosure - Mothers whose male partner or family members were informed of their HIV test results [21].

No formal education – HIV-positive women who cannot read and write.

Woreda - An administrative division of Ethiopia managed by local government, woredas are composed of several kebeles or neighbourhoods’ associations, which are the smallest units of local government in Ethiopia. Woredas are typically grouped into zones.

### 2.10 Data collection tools and procedures

A structured and standardized questionnaire was used to collect data through face-to-face interviews [28]. To ensure question consistency, the questionnaire was initially written in English, translated into Amharic (Agewugna), and then back translated into English by language specialists.

To evaluate the patients' medical circumstances, such as the date of HIV diagnosis, start of therapy, CD4 count upon admission, and WHO clinical stage, respondents were interviewed, and patient records were examined. Nine midwives with diplomas served as data collectors, while five midwives with bachelor's degrees served as supervisors during the distribution of a structured questionnaire.

### 2.11 Data quality control

The questionnaire was pretested at the Chara Center, which was not included in the study, using 5 % of the sample population. This was done to ensure uniformity in terms of organization, coherence, thoroughness, and ease of understanding of the questions. Following the pretest, the questionnaire was appropriately updated.

Before beginning the actual data gathering process, the data collectors received training on sampling and data collection techniques. Each day, supervisors and investigators checked and reviewed the completed questionnaires to ensure that the data were accurate and consistent.

### 2.12 Data processing and analysis

After being coded, cleaned, and imported into EPI-Data version 3.1, the data were exported to SPSS version 25 for further examination. The data were summarized through descriptive analysis, including frequency tables and graph.

Bivariable analysis was conducted to examine the relationship between each explanatory variable and the dependent variable. To control for confounding variables, only those significantly correlated with the outcome variable in bivariate regression were included in the multivariable logistic regression analysis. Variables demonstrating a relationship with the dependent variable at a P-value less than 20 % in the bivariate analysis were added to the multivariate logistic regression analysis. This analysis aimed to identify correlations with the dependent variable. An adjusted odds ratio and a significance level of ≤0.05, along with a 95 % confidence interval, were used to indicate a statistically significant relationship between the independent predictors and the outcome variable. The goodness of fit of the final model was assessed using the Hosmer–Lemeshow goodness-of-fit test.

## 3 Results

### 3.1 Sociodemographic characteristics of the respondents

The study included a total of 358 HIV-positive women, all of whom participated, for a response rate of 100 %. Among all the participants, 113 (37.5 %) were aged between 18 and 30 years, with a mean age of 29.29 years and a standard deviation of approximately ±4.44 years. Additionally, 261 (72.9 %) and 285 (79.6 %) of the total respondents were married and had received formal education, respectively (Table 2).

**Table 2:** Sociodemographic characteristics of the respondents regarding adherence to option B + PMTCT in the Awi zone, northwestern Ethiopia, March 1-April 30, 2020.

Variables	Frequency(N = 358)	Percentage (%)
**Age in groups**		
18–24	43	12.0
25–29	138	38.5
30–34	128	35.8
35 and above	49	13.7
**Place of residence**		
Rular	60	16.8
Urban	298	83.2
**Religion**		
Orthodox	315	88.0
Muslim	37	10.3
Protestant	6	1.7
**Marital status**		
Single	60	16.8
Married	261	72.9
Divorced	35	9.8
Windowed	2	0.5
**Educational level**		
Have formal education	285	79.6
No formal education	73	20.4
**Occupatio**n		
Merchant	136	38.0
Governmental employee	51	14.2
Housewife	158	44.1
Private employee	13	3.7
**Types of health facilities**		
Health center	225	62.8
Hospital	133	37.2
**Time is taken to reach home to the facility on foot**		
Less than 1 h	302	84.4
Greater than 1 h	56	15.6

## 4 Disclosure of HIV status among HIV-positive women

In this study, approximately 323 (90.2 %) of the participants disclosed their HIV sero status (79.6 % of them disclosed to their partner, 6.2 % of them to their friends, 3.2 % of them to their family, and 0.6 % of them disclosed to others) (Table 3).

**Table 3:** HIV disclosure status of respondents in selected governmental health facilities in the Awi zone, northwestern Ethiopia, March 1-April 30, 2020.

Variables	Frequency(N = 358)	Percentage (%))
Disclosed your HIV sero status to others		
Yes	323	90.2
No	35	9.8
**For whom you disclosed** (n = 323)		
Partner	257	79.6
Friend	20	6.2
Family	44	13.6
Others	2	0.6
**Partner/family/help you to take your medication regularly and on time**		
Yes	312	96.6
No	11	3.4
**Partner HIV status**		
Positive	299	83.5
Negative	39	10.9
Don't know	20	5.6
**Partner on medication**(n = 319)		
Yes	288	90.3
No	11	3.4
Don't know	20	6.3

### 4.1 HIV AIDS treatment-related characteristics of the respondents

When participants were admitted to the option B+ program, most of the respondents were in stage 1. At admission, 304 (84.9 %) of the total respondents had a CD4 count above 350 cells/mm3. Most respondents (351; 98 %) received counselling regarding the adverse effects of ART drugs. However, 273 (76.3 %) of the women were counselled for less than 30 min (Table 4).

**Table 4:** HIV AIDS-treatment-related characteristics among the respondents in selected governmental health facilities in the Awi zone, northwestern Ethiopia, March 1-April 30, 2020.

Variables	Frequency(n = 358)	Percentage (%)
**WHO clinical category at admission in option B plus**		
Stage one	305	85.2
Stage two	44	12.3
Stage Three	9	2.5
**WHO clinical category during the study period**		
Stage one	354	98.9
Stage two	4	1.1
**Started ART with CD4 count or clinical stage**		
Yes	56	15.6
No	302	84.4
**CD4 count at admission**		
<350	54	15.1
≥350	304	84.9
**Time of initiation of option B + PMTCT drug**		
Pregnancy	106	29.6
Breastfeeding	6	1.7
Previously known	246	68.7
HIV testing and taking ART treatment on the same day of diagnosis is challenging		
Yes	68	19.0
No	290	81.0
**When you know HIV status at PMTCT enrolment**		
Known before pregnancy at the ART clinic.	276	77.1
Known during pregnancy and breastfeeding at the PMTCT clinic	82	22.9
**Have you received counselling about the side effect of ART drugs?**		
Yes	333	93.0
No	25	7.0
**Duration of counselling**		
<30 min	273	76.3
≥30 min	85	23.7
**Have you experienced any side effects from ART drugs?**		
Yes	62	17.3
No	296	82.7
**The side effect of option B plus PMTCT ART drugs**(n = 62)		
Nausea	15	24.2
Vomiting	22	35.5
Dizziness	23	37.1
Skin rash	2	3.2

### 4.2 Male partner involvement in option B + PMTCT drug adherence

Regarding male partner involvement in option B + PMTCT drugs, 240 (67.04 %) of the respondents had good partner support, 83 (23.26 %) of the respondents had moderate levels of partner support, and 35 (9.5 %) of the respondents had low partner support (Table 5).

**Table 5:** Male partner involvement in option B + PMTCT drug adherence in selected government health facilities in the Awi zone, Northwestern Ethiopia, March 1-April 30, 2020.

Male partner involvement	Percentage and frequency	
	Yes	No
Shares his wife's decisions on household issues	265(74.0)	93(26.0)
Discusses with his wife on use of condoms during sex	181(50.6)	177(49.4)
Knows the frequency of taking PMTCT drug	317(88.5)	41(11.5)
Visits the PMTCT clinic with his wife to bring ARV drugs	121(33.8)	237(66.2)
Knows the name of PMTCT drugs	224(62.6)	134(37.4)
Knows the doses of PMTCT drugs	196(54.7)	162(45.3)
Discusses the advantages of ANC/PNC appointment	278(77.7)	80(22.3)
Supports his wife financially to visit ANC/PNC PMTCT	284(79.3)	74(20.7)
Attend his wife's PMTCT follow up	319(89.1)	39(10.9)
Reminds his wife about ANC/PNC appointment	318(88.8)	40(11.2)
Level of partner support		
Good	240 (67.0)	
Moderate	84 (23.3)	
Low	34 (9.5)	

### 4.3 Knowledge regarding option B + PMTCT among HIV-positive women

In this study, 307 (85.8 %) of the respondents reported taking ART drugs to minimize the risk of HIV transmission to their infants. Regarding their understanding of option B plus PMTCT drug adherence, 283 (79.1 %) participants possessed a high level of knowledge, 66 (18.4 %) had a moderate level, and 9 (2.5 %) had a low level (Table 6).

**Table 6:** Knowledge questions regarding Option B + PMTCT drug adherence among the respondents in the Awi zone of selected government health facilities in Northwest Ethiopia, March 1-April 30, 2020.

Characteristics	Frequency(n = 358)	Percentage (%)
**Do you know how HIV is transmitted?**		
Yes	355	99.2
No	3	0.8
Can pregnant and breastfeeding women be living with HIV AIDS transmit the disease to her baby		
Yes	355	99.2
No	3	0.8
Have you ever heard about lifelong treatment of ART for any HIV-positive pregnant & breastfeeding mothers		
Yes	331	92.5
No	27	7.5
Condom use can prevent HIV transmission during sex with an HIV infected partner		
Yes	310	86.6
No	48	13.4
HIV-positive women can reduce the risk of HIV transmission to their babies if they take PMTCT drugs		
Yes	307	85.8
No	51	14.2
Omitting to take some of the PMTCT drugs' effects on the effectiveness of PMTCT care and support		
Yes	285	79.6
No	73	20.4
reduce the risk of opportunistic Adhering to ARV drugs can infections		
Yes	274	76.5
No	84	23.5
The support of the male partner during PMTCT care does not have any effect on mothers adhering to PMTCT drug		
Yes	102	28.5
No	256	71.5
**Level of knowledge**		
Low	9	2.5
Moderate	66	18.4
High	283	79.1

### 4.4 Level of option B + PMTCT drug adherence

In this study, the rate of adherence to option B + PMTCT drugs among HIV-positive women was 83.24 %, while 16.76 % of the respondents did not adhere (Table 7).

**Table 7:** Respondents' level of adherence to ward option B + PMTCT in selected governmental health facilities in the Awi zone, Northwestern Ethiopia, March 1-April 30, 2020.

Characteristics	Frequency and percentage	
	No	Yes
Do you sometimes find it difficult to remember to take your Medication	321(89.7)	37(10.3)
When you feel better, do you sometimes stop taking Your medication?	340(95.0)	18(5.0)
Many patients have trouble taking their ARV doses As prescribed; did you miss any ARV doses in the last 3 days	345(96.4)	13(3.6)
Sometimes if you feel worse when you take the medicine, Do you stop taking it	338(94.4)	20(5.6)
Level of option B plus adherence		
Good	298(83.2)	
Poor	60(16.8)	

### 4.5 Challenges of option B plus PMTCT drug adherence

**Fig. 2:**
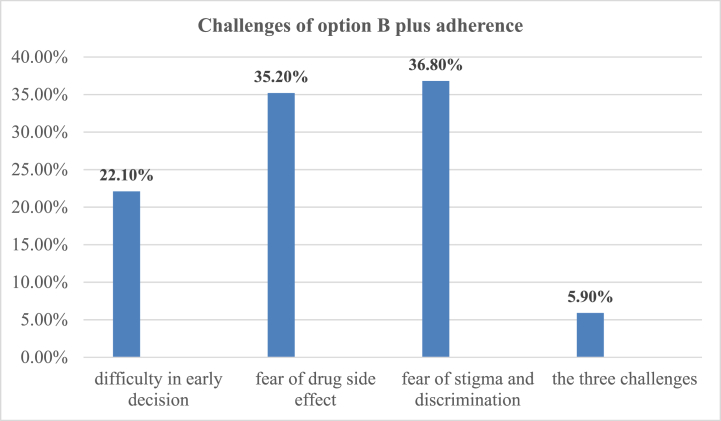
Challenge of option B + PMTCT drug adherence among the respondents in selected health facility of Awi zone, North West Ethiopia, March 1-April 30, 2020(N = 358).

The primary challenges to adherence to option B + PMTCT drugs were fear of stigma and discrimination, accounting for 36.8 %, followed by concerns about drug side effects, at 35.2 % (Fig. 2)

### 4.6 Factors associated with adherence to option B + PMTCT

Bivariate logistic regression analysis indicated that educational status, initiation of Option B + PMTCT drugs, partner support, good counselling, time taken to reach from home to health facilities, and challenges with HIV testing and taking ART treatment on the day of diagnosis were significantly associated with adherence to Option B + PMTCT drugs. However, in multivariable logistic regression analysis, partner support, counselling, and time taken to reach from home to the facility were shown to be significantly associated with the level of adherence to Option B + PMTCT drugs.

Participants who had good partner support involvement were 3.0 times more likely to adhere to Option B + PMTCT drugs than those who had a low level of partner support involvement [AOR = 3.0, 95 % CI (1.17–7.92)].

Participants who travelled less than 60 min for Option B+ services were 3.1 times more likely to adhere to Option B + PMTCT drugs than those who travelled 60 min or more for Option B+ services [AOR = 3.1, 95 % CI: (1.51–6.52)].

The study participants who received counselling were 4.4 times more likely to adhere to option B, the PMTCT drug, than participants who did not receive good counselling [AOR = 4.4 (95 % CI = 1.60–12.29)] (Table 8).

**Table 8:** Bivariate and multivariate logistic regression analyses of factors associated with adherence to Option B + PMTCT among respondents in selected government health facilities in the Awi zone in Northwest Ethiopia from March 1-April 30, 2020.

	Adherence level		Odds Ratio		P- valve
Variables	Good n (%)	Poor n (%)	COR (95%CI)	AOR (95 % CI)	
Educational level					
Formal education	249(83.6)	36(60.0)	3.4(1.86–6.18)	2.5(0.52–12.02)	0.09
No formal education	49(16.4)	24(40.0)	1	1	1
partner support involvement					
Good	216(72.5)	24(40.0)	3.7(1.60 8.77)	3.0(1.17–7.92)	0.02*
Moderate	58(19.5)	26(43.3)	0.9(0.39–2.22)	0.95(0.35–2.57)	0.93
Low	24(8.0)	10(16.7)	1	1	**1**
Time is taken to reach from home to facility					
<60 min	265(88.9)	37(61.7)	4.9(2.65–9.41)	3.1 (1.51–6.52)	0.002*
≥60 min	33(11.1)	23(38.3)	1	1	1
Initiation of option B + PMTCT drug					
Breastfeed	5(1.7)	1(1.6)	2(0.232–18.42)	3.4(0.30–39.40)	0.33
Previously	218(73.1)	28(46.7)	3.2(1.81–5.76)	2.4(0.92–6.26)	0.07
Pregnancy	75(25.2)	31(51.7)	1	1	1
Counselling about the side effect of ART drug					
Yes	283(95.0)	50(83.3)	3.8(1.61–8.87)	4.4(1.60–12.29**)**	0.04*
No	15(5.0)	10(16.7)	1	1	1
HIV testing and taking ART treatment on the same day of diagnosis is challenging					
Yes	50(16.8)	18(30.0)	0.47(0.25–0.88)	0.9(0.41–1.88)	0.73
No	248(83.2)	42(70.0)	1	1	1

A P value ≤ 0.05 was considered to indicate statistical significance**.**

## 5 Discussion

Our findings revealed that 83.24 % of the participants adhered to Option B + PMTCT medication. These results align with previous cross-sectional studies conducted in southern Ethiopia [30], the Hadiya zone [24], and the East Shewa zone in Ethiopia [31], where adherence rates to Option B + PMTCT medication were reported to be 81.4 %, 83.7 %, and 82.6 %, respectively.

This consistency in adherence rates could be attributed to similarities in the sociodemographic characteristics of the participants. For instance, in both Hadiya and southern Ethiopia, a substantial proportion of respondents fell within the 25–29 age group, accounting for 47 % and 39.3 %, respectively. Additionally, the utilization of a self-reported adherence measurement tool, the study design, and the rapid expansion and implementation of the Option B+ program at the national level might have contributed to these findings.

Furthermore, our study findings are consistent with those of a cross-sectional study conducted in Lagos, Nigeria (80.6 %) and Mugalo Hospital in Uganda (86 %), where adherence to Option B + PMTCT medication was reported [32,33]. This agreement could stem from similarities in the adherence measurement tool, both utilizing self-reporting methods, as well as similarities in study design. Additionally, shared sociodemographic characteristics among respondents might also play a role. For example, in Lagos, Nigeria, a significant portion of respondents were married (77.1 %), which could contribute to the observed consistency in adherence rates.

In contrast to studies conducted in Kenya (61.9 %) [34], Nigeria (78.3 %) [35], and the United States of America (70 %) [36], the adherence level in this study was higher. This discrepancy may be attributed to differences in the PMTCT approach used. While these studies focused on Option A PMTCT, our study examined Option B+. Option B+ is considered a more straightforward approach, which could have influenced the adherence levels observed.

Moreover, these findings were lower than those of other studies conducted in South Wollo (87.9 %) [23] and in Mekelle, Tigray (87.1 % [29]. This variation could be attributed to differences in the study settings and adherence measurement tools. Unlike the study conducted in Mekelle town, our study incorporated data from both hospitals and health centers, providing a broader perspective on Option B + PMTCT adherence. Additionally, while the study in South Wollo Zone used pill count and self-reporting methods for adherence measurement, our study solely relied on self-reporting adherence measurement tools, offering a different approach to assessing medication adherence in PMTCT programs. Research suggests that women receiving care at primary healthcare facilities, such as health centers, may exhibit lower adherence to ART medications than those receiving care at hospitals [32].This is because hospitals often have more resources and infrastructure, which allows for better counselling services that can positively impact adherence to ART medications.

This result is also lower than that of a study conducted in western Uganda, where the median adherence to Option B+ during pregnancy was 95.7 % [26]. The difference in adherence rates between this study and the one conducted in western Uganda could be attributed to variations in the adherence assessment methods employed. Additionally, the Ugandan study specifically focused on pregnant women, which might have influenced the reported adherence rates. Discrepancies could also arise from differences in sociodemographic factors. For instance, in western Uganda, a higher proportion (52.4 %) of respondents fell within the 30–34 age group, whereas in our study, the majority (38.5 %) were aged between 25 and 29.

The findings of this study revealed that participants who received good support from their partners were three times more likely to adhere to Option B + PMTCT medication than were those with low partner involvement. These findings are consistent with research conducted in Tanzania [37], Lilongwe in Malawi [27], and Mugalo Hospital in Uganda [33]. This is because having a supportive partner fosters an environment conducive to adherence to the PMTCT drug regimen for HIV-positive women. Emotional support, financial assistance, reminders to take medications, encouragement to seek prenatal care, and overall partner encouragement positively impact a woman's motivation and ability to consistently follow the treatment plan [21].

Participants who travelled less than 60 min were 3.1 times more likely to adhere to Option B + PMTCT medication. These findings align with those of a cross-sectional study conducted in southern Ethiopia [30].

The findings of this study were also supported by studies performed in Nepal [38] and the WHO [10]. This could be due to shorter travel times improving accessibility to healthcare facilities, facilitating timely refills and medication adherence. Additionally, reduced travel time may alleviate the financial and logistical burdens associated with transportation, thereby enabling women to consistently access and adhere to their PMTCT medication. Fear of discrimination and stigma emerged as major barriers to Option B + PMTCT adherence in the study, affecting 36.8 % of participants and 35.5 % of respondents, respectively. Consequently, HIV-positive pregnant or breastfeeding women might choose to seek follow-up care outside their local area to address these challenges.

The findings of this study also indicated that participants who were counselled about option B + PMTCT drug were 4.4 times more likely to adhere to option B + PMTCT. These findings were supported by studies conducted in East Shewa [31] and in the Hadiya zone in Ethiopia [24].This finding was also supported by a study performed in Ukraine [25]. This might be due to effective counselling, which offers information, support, and motivation, helping individuals overcome adherence barriers and adhere to their treatment regimen. Additionally, HIV-positive women who receive counselling about drug side effects may exhibit better adherence to Option B + PMTCT medication [21].

### 5.1 Strengths and limitations of the study

This study has the strength of identifying multiple factors associated with adherence to the Option B + PMTCT program. Another strength of this study was the inclusion of many health facilities, such as four hospitals and five health centers, and achieving a 100 % response rate from the respondents, which strengthens the reliability of the collected data. Given that the Option B+ program was recently implemented in our country, it is important to assess the level of adherence across many health facilities.

Due to logistical and time constraints, this study exclusively utilized a quantitative approach, thereby limiting its ability to comprehensively address 'why' questions.

Adherence categorization may be susceptible to recall bias, despite attempts to minimize it through strategies such as conducting interviews in private rooms, using prompting techniques, employing memory aids, asking for concrete examples, and encouraging participants to provide specific instances related to the topic of interest. While social desirability bias could also affect the study, efforts were made to mitigate it. Participants were briefed on the study's purpose, confidentiality measures, and the importance of honest participation, which potentially influenced the study's outcomes. Additionally, adherence was subjectively measured. To alleviate this, clear instructions were provided, confidentiality was ensured, leading questions were avoided, participants were educated on the importance of accurate reporting and the utilization of their adherence data, data quality was monitored, structured interviews were conducted, and existing validated tools from South Africa were utilized.

## 6 Conclusion and recommendations

### 6.1 Conclusion

This study revealed that the level of Option B + PMTCT drug adherence was lower than the nationally recommended adherence level. Partner support, counselling, and the time taken to reach from home to the health facility were significantly associated with the level of option B + PMTCT drug adherence.

### 6.2 Recommendation

Currently, information, education, and communication (IEC) interventions for HIV/AIDS-positive women should be strengthened at the individual and community levels to minimize the distance between the health center and the individual's residence, thereby enhancing adherence to Option B plus PMTCT drugs. When health facilities are easily accessible, the burden of travel is reduced, and regular clinic visits for medication refills and check-ups are encouraged. This convenience can help individuals stay on track with their treatment plan and improve overall adherence to PMTCT drugs. If distance proves to be a barrier, alternative solutions such as mobile clinics, community health workers, or medication delivery services may be considered to support adherence.

It is important to provide comprehensive and tailored counselling for individuals on Option B PMTCT drug adherence. Counselling should emphasize medication adherence to prevent HIV transmission, address concerns, and highlight treatment benefits. It should also involve the family and provide ongoing support for positive health outcomes.

Partners can aid by reminding individuals to take their medication, attending appointments with them, and offering emotional support. Partner involvement, characterized by understanding and active participation, promotes adherence and enhances health outcomes for both mother and child. Subsequent research should be conducted using a longitudinal approach in conjunction with a qualitative study design.

## Ethics approval and consent to participate

Ethical approval for this study was obtained from the Bahir Dar University institutional review board; the reference number of the letter was BDU/00195/2020. A formal letter of support was obtained from the Awi Zone HealthOffice.

Written informed consent was obtained from the individual participants. Moreover, the purpose, procedures of the study, advantages, and disadvantages of the study were described to the participants. All the participants in the study participated voluntarily, and their information was kept confidential. Participants were informed that they had the right to the data drawn at any time.

## Funding

This research was supported by a grant from 10.13039/501100005872Bahir Dar University, College of Medicine, and 10.13039/100018491Health Sciences School of 10.13039/100018696Health Science. The granting agency did not have a role in the design; collection, analysis, or interpretation of the data; or writing of the manuscript.

## Data availability

The data included in the article/supplementary material/referenced in the article. No data associated with our study have been deposited into a publicly available repository.

## CRediT authorship contribution statement

**Tegegne Wale Belachew:** Writing – review & editing, Writing – original draft, Validation, Supervision, Software, Methodology, Investigation, Formal analysis, Data curation, Conceptualization. **Besfat Berihun Erega:** Writing – review & editing, Writing – original draft, Validation, Supervision, Methodology. **Mesafint Ewunetu:** Writing – review & editing, Writing – original draft, Visualization, Validation, Supervision, Software, Methodology, Formal analysis, Conceptualization. **Kihinetu Gelaye:** Writing – review & editing, Writing – original draft, Validation, Supervision, Software, Methodology, Formal analysis, Conceptualization. **Tigist Seid Yimer:** Writing – review & editing, Writing – original draft, Software, Methodology, Data curation. **Wassie Yazie Ferede:** Writing – review & editing, Writing – original draft, Visualization, Validation, Supervision, Software, Methodology, Formal analysis, Conceptualization.

## Declaration of competing interest

The authors declare the following financial interests/personal relationships which may be considered as potential competing interests: Tegegne Wale Belachew reports was provided by Debre Tabor university. Tegegne Wale Belachew reports a relationship with Debre Tabor University that includes: employment. Tegegne Wale Belachew has patent pending to Yes. Wassie Yazie Ferede If there are other authors, they declare that they have no known competing financial interests or personal relationships that could have appeared to influence the work reported in this paper.

## List of abbreviations and acronyms

AIDS: Acquired Immune Deficiency Syndrome
AOR: Adjusted Odd Ratio
ANC: Antenatal Care
ART: Anti-Retro Viral Therapy
ARV: Anti-Retro Viral
CI: Confidence Interval
EMTCT: Elimination of Mother to Child Transmission
HIV: Human Immune Virus
MTCT: Mother-to-Child Transmission
PMTCT: Prevention of Mother to Child Transmission
