# Supplementary material for: Level of option B plus drug adherence for preventing mother-to-child transmission of HIV and associated factors among HIV-positive women in the awi zone, amhara region, northwest Ethiopia,2020
Source: Heliyon. 2024 Jul 26;10(15):e35319. doi: 10.1016/j.heliyon.2024.e35319 (PMC11332886; doi:10.1016/j.heliyon.2024.e35319)
Supplement: Multimedia component 1 [file mmc1.docx]

# **Annexes**

**Annex I: Information& consent form**

Good morning or afternoon, my name is …………………. I am working as a data collector in a study conducted by Tegegne Wale in the midwifery department, school of health science, college of medicine, and health science at Bahir Dar University for a second degree. The objective of this study is to assess the level of adherence and associated factors to option B+ PMTCT drugs among pregnant and breast-feeding mothers in selected government health facilities in the Awi Zone, North West Ethiopia. I am interviewing HIV-positive pregnant and breast-feeding mothers until 18 months who are on option B plus PMTCT service. So, your cooperation has played a great role in the fruitfulness of this study. You do not have to answer any questions that you do not want to answer, and you may end this interview at any time you want. Any care or service provision will never be discontinued related to your refusal to participate in this study. The care and support will continue even if you don’t accept this study. You can change your idea at any time, even if you accept the study.

**Risks:** By participating in this study, you will not face any risk, but if you suspect any risk or have any questions, you can raise them at any time.

**Benefits and incentives:** There are no incentives you get from participating in this study. But the aim of this study is to identify any children not born with HIV virus.

**Confidentiality:** Your information will not be disclosed to anyone except by the investigator. Your name will not be written on the paper, but only by coding. This code is known only to the data collector. The data may be seen by investigators, advisors, and data collectors, but for others, it will not be disclosed.

**Share the findings:** If you want to know the findings at the end of the study, we can communicate. The result of the study will be disseminated to different scholars, scientific communities, and others who want to know.

**Time of interview**: The interview will take about 20-30 minutes

If you have questions about this study, you can ask them.

Principal investigator: Tegegne wale

Mobile number. +252918581910/email [tegegnewale10@gmial.com](mailto:tegegnewale10@gmial.com)

Would you Participant in our study? A. Yes B. No

If yes continue or if no give thanks & precede to other participant

Signature of participate

Date of interview … Name of data collector…………… Date ……./……….Signature…………..

**Annex II: English version questionnaire**

**Part 1: Questions on sociodemographic characteristics of participants in selected government health facilities of Awi zone**

| s.no | Variables | Response | Skip to |
| --- | --- | --- | --- |
| 101 | Age in years | ---------------------------- |  |
| 102 | Place of residence | Rural |  |
|  |  | Urban |  |
| 103 | What is your religion? | Orthodox Christian |  |
|  |  | Muslim |  |
|  |  | Protestant Christian |  |
|  |  | Catholic |  |
| 104 | What is your marital status? | Single |  |
|  |  | Married |  |
|  |  | Divorced |  |
|  |  | Windowed |  |
| 105 | What is your level of education? | No formal education |  |
|  |  | Primary (grade1-8) |  |
|  |  | Secondary(grade 9-12) |  |
|  |  | Diploma |  |
|  |  | Technical/vocational |  |
|  |  | Degree and above |  |
| 106 | What is your occupation currently? | Housewife |  |
|  |  | Merchant |  |
|  |  | Private employee |  |
|  |  | Governmental employee |  |
|  |  | Student |  |
|  |  | Other(specify) |  |
| 107 | Types of health facilities | …………………………. |  |
|  | Time is taken to reach home to the facility on foot |  |  |

**Part 2: HIV status disclosure among pregnant & breastfeeding mothers until 18 months in selected governmental health facilities of Awi zone**

| s.no | Question | Response | Skip to |
| --- | --- | --- | --- |
| 201 | Have you disclosed your status to anyone? | Yes  No | If no go  to qn.204 |
| 202 | For Whom did you disclose your status? | Partner  Family  friend other___ |  |
| 203 | Did they help you take your pills regularly  on time? | Yes  No |  |
| 204 | Is your partner HIV positive? | Yes  No  Don’t know |  |
| 205 | Is He on medication | Yes  No  Don’t know |  |

**Part 3: HIV disease & treatment related questions among pregnant & breastfeeding mothers until 18 months in selected governmental health facilities of Awi zone**

| s.no | Questions | Response | Skip to |
| --- | --- | --- | --- |
| 301 | WHO clinical category at admission? | Stage 1  Stage 2  Stage 3  Stage 4 | Review  Patient  Medical  record |
| 302 | WHO clinical category during the study time | Stage 1  Stage 2 |  |
| 303 | Did you start your ART with CD4 count or clinical stage? | Yes  No |  |
| 304 | CD4 count at admission, cells/mm3 | ……………………………. | Review  Card |
| 305 | When you started your option B+ drugs? | During this pregnancy  During breastfeeding  Previously known |  |
| 306 | Do you think HIV testing & taking ARV treatment at the same  day of HIV diagnosis is challenging? | yes  No | If no  Skip to  q.n 308 |
| 307 | What are the challenges? | Difficulty in early  decision,  Fear of side effects of  drug,  Fear of stigma,  difficult To  disclose to partner  Others_____ |  |
| 308 | HIV status at PMTCT enrolment | Newly diagnosed  during breastfeeding |  |
|  |  | Already known HIV  Status |  |
|  |  | Newly diagnosed  during pregnancy  pregnancy |  |
| 309 | Did you counsel about the side effect of ART drug? | Yes  No |  |
| 310 | How long have you counseled |  |  |
| 311 | Have you experienced any side effects from the medicines  You are taking? | Yes  No | If no  skip  q.n 401 |
| 312 | What did you experience? | Nausea  Vomiting  Dizziness  Skin rash  Other_____ |  |
| 313 | Were the medicine(s) changed for you  After experiencing side effects? | Yes  No |  |

**Part 4: Male partner involvement towards option B+ PMTCT drugs in selected government health facilities of Awi zone, North West Ethiopia,**

| s.no | Male involvement activities | Yes | No |
| --- | --- | --- | --- |
| 401 | Shares his wife's decisions on household issues |  |  |
| 402 | Discusses with his wife on use of condoms during sex |  |  |
| 403 | Knows the frequency of taking PMTCT drug |  |  |
| 404 | Visits the PMTCT clinic with his wife to bring ARV drugs |  |  |
| 405 | Knows the name of PMTCT drugs |  |  |
| 406 | Knows the doses of PMTCT drugs |  |  |
| 407 | Discusses the advantages of ANC/PNC appointment |  |  |
| 408 | Supports his wife ﬁnancially to visit ANC/PNC PMTCT |  |  |
| 409 | Attends PMTCT with his wife |  |  |
| 410 | Reminds his wife about ANC/PNC appointment |  |  |

**Part 5: Knowledge questions regarding Option B+ PMTCT among pregnant & breastfeeding mothers until 18 months in selected government health facilities Awi zone**

| s.no | Characteristics | Response | Skip to |
| --- | --- | --- | --- |
| 501 | Do you know how HIV is transmitted? | Yes  No |  |
| 502 | Is there any means to avoid transmission of HIV from the mother to her child | Yes  No |  |
| 503 | Have you ever heard about life long treatment ofART for any HIV positive  pregnant & breastfeeding mothers | Yes  No |  |
| 504 | Condom use can prevent HIV transmission during sex with an HIV-infected partner | yes  no |  |
| 505 | HIV-positive women can reduce the risk of HIV transmission to their babies if  they take PMTCT drugs | yes  no |  |
| 506 | Omitting to take some of the PMTCT drugs Affects the effectiveness of  PMTCTcare and support | yes  no |  |
| 507 | Adhering to ARV drugs can reduce the risk of opportunistic infections | yes  no |  |
| 508 | The support of a male partner during PMTCT care does not have any effect on  mothers adhering to PMTCT drugs | yes  no |  |

**Part 6: Assessment of level of adherence to option B+ PMTCT drugs among pregnant & breastfeeding mothers in selected governmental health facilities of Awi zone by Self-reporting questions**

| s.no | Questions | Response | Skip to |
| --- | --- | --- | --- |
| 601 | Do you sometimes ﬁnd it difficult to remember to take your Medication? | Yes  No |  |
| 602 | When you feel better, do you sometimes stop taking Your medication? | Yes  No |  |
| 603 | Many patients have trouble taking their ARV doses As prescribed;  did you miss any ARV doses in the last 3 days? | Yes  No |  |
| 604 | Sometimes if you feel worse when you take the medicine, Do you stop taking it? | Yes  No |  |

**Annex III: በአማረኛ የተተረጎመ የስምምነት እና መረጃ ቅፅ**

በአዉይ ዞን በተመረጡ የመንግስት ጤና ተቋማት ዉስጥ የፀረ ኤች ኤይቪ መድሃኒት የሚጠቀሙ ነፍሰጡር እና ጡት ለሚያጠቡ እናቶች የተዘጋጀ ቃለመጠይቅ 01-ቀን / -- / 02-የጥያቄዎች መለያ ቁጥር_______03 የጤና ተቋም ስም- **የመረጃ መግለጪያ ቅፅ እና ስምምነት**

ስሜ--------------እባላለሁ፡፡ በባህርዳር ዩኒቨርሲቲ ሕክምና ፋኩሊቲ ሚድዋይፈሪ ት/ት ክፍል በሚደረገው ጥናት ላይ ማለትም ኤች ኤይቪ ኤድስ ከእናት ወደ ልጅ እንዳይተላለፍ በሚደረገዉ ህክምና ላይ የኤች ኤይቪ ኤድስ ቫይረሱ በደማቸዉ ዉስጥ ለሚገኝባቸዉ ነፍሰ ጡር እና የሚያጠቡ እናቶች ያለምንም ቅድመ ሁኔታ(የሲዲፎር መጠን እና የበሽታዉ ደረጃ) ለህይወት ዘመን የሚወሰድ የፀረ ኤች ኤይ ቪ ኤድስ መድሃኒት ማስጀመር በሚባለዉ ምርጫ ላይ እናቶች ከመድሃኒቱ ጋር ያላቸው ቁርኝት እና አወሳሰድ እንዲሁም ሌሎች ተያያዥ ነገሮችን የሚዳስስ መረጃዎችን ለመሰብሰብ ነው፡፡ በመሆኑም የእርስዎ ትብብር ለጥናቱ መሳካት የማይተካ ሚና አለው፡፡ በዚህ ጥናት ላይ መሳተፍ ካልፈለጉ በአገልግሎት አሰጣጡ ላይ ምንም የሚለወጥ ነገር አይኖርም፡፡ የሚሰጠዉ እንክብካቤ ይቀጥላል፡፡አሁን አዎ ቢሉም እንኳን ሃሳብዎን መቀየር ይችላሉ፡፡

**ስጋቶች**፤እርስዎ የጥናቱ ባለቤት በመሆንዎ የሚያሰጋዎት ምንም ነገር አይኖርም፡፡ነገር ግን ያልተለመደ ነገር ካጋጠመዎ በማንኛዉም ስዓት ነፃ ሆነዉ ያለዎትን ስጋትና ጥያቄ እንዲነግሩን እንፈልጋለን፡፡

**ጥቅም እና ማበረታቻ፡** በዚህ ጥናት በመሳተፍዎ የሚሰጥዎት ጥቅም ሆነ ማበረታቻ የለም፡፡ነገር ግን ይህ ጥናት ማንኛዉም ህፃን ከኤች ኤይቪ ኤድስ ጋር መወለድ የለበትም የሚለዉን እራዕይ ለማሳካት ከፍተኛ ሚና አለዉ፡፡

**ሚስጥር መጠበቅ፡** ከእርስዎ የሚሰበሰበዉ መረጃ በሚስጥርነት የሚያዝና ከአጥኝዉ በስተቀር ሌላ ሰዉ አያየዉም፡፡ማንኛዉም ስለእርስዎ የሚሰበሰብ መረጃ ላይ ስምዎ አይፃፍም፡፡ በስምዎ ምትክ የሚስጥር ቁጥር የሚሰጠዉ ይሆናል፡፡ ይህን ቁጥር የሚያዉቀዉ አጥኝዉ ብቻነዉ፡፡ ከአጥኝዉ ከአማካሪዉ ከጥናቱ ስፖንሰር አድራጊ በስተቀር መረጃዉ ለማንም ሌላ ሰዉ ተላልፎ አይሰጥም፡፡ **ግኝቶችን ስለመግለፅ፡** እርስዎ የጥናቱን ግኝት ማወቅ ከፈለጉ ጥናቱ ካለቀ በኋላ ከእርስዎ ጋር መወያየት እንችላለን እንዲሁም ስለጥናቱዉት ለሌሎች ምሁራን እና ለተለያዩ ህብረተሰብ የምናካፍል ይሆናል፡፡ይህን የምናደርገዉ የጥናቱን ዉጤት ለማወቅ ለሚፈልጉ ሁሉ በፅሁፍ እና በተለያዩ ድህረ-ገፅ ነዉ፡፡ ጥናቱ የሚወስደዉ ግዜ - ይህ ጥናት ከ 20 ደቂቃ እስከ 30 ደቂቃ ያህል ግዜ ይወስዳል **፡፡ የጥናቱ ተሳታፊ የስምምነት መረጃ**

ከላይ የተገለፀልኝን መረጃ በትከክል አዳምጬ ተረድቻለሁ፡፡ የጥናቱን ትቅም፤የሚያመጣዉ ችግር፤ሚስጥር መጠበቅ፡ ጥቅማጥቅም እና የሚወስደዉ ግዜ በተመለከተ ተገንዝቤአለሁ፡፡

ይህን ጥናት በተመለከተ ማነጋገር ወይም መጠየቅ የሚፈልጉት ጉዳይ ካለዎት የዋና አጥኝዉ አድራሻ እንደሚከተለዉ እገልፃለን

ስም፡ ተገኘ ዋለ ሰልክ ቁጥር- 0918581910 ኢሜይል [tegegewale10@gmail.com](mailto:tegegewale10@gmail.com)

በጥናቱ ላይ ለመሳተፍ ፈቃደኛ ነሽ? ሀ.አዎ ለ. አልፈልግም

አዎ ከሆነ ይቀጥሉ፤አልፈልግም ካሉ አመስግኑ እና ወደ ሚቀጥለዉ ቃለመጠይቅ ይሂዱ፡፡

የመረጃ ሰብሳቢዉ ስም --------------------------ፊርማ--------------ቀን-------------

**Annex IV*:የጥናቱ መጠይቆች***

**ክፍል1: ማሕበራዊ ፤ አካባቢያዊና ኢኮኖሚያዊ ጋር የተያያዙ ጥያቄዎች፡ ፡ በአዊ ዞን በተመረጡ የመንግስት ጤና ተቋማት ለሚገኙ ኤች አይቪ ቫይረስ በደማቸዉ ለሚገኝባቸዉ ነፍሰ ጡር እና የሚያጠቡ እናቶች የተዘጋጀ**

| ተ.ቁ | ጥያቄዎች | መልስ | ዝለል |
| --- | --- | --- | --- |
| 101 | እድሜዎት ስንት ነው? | --------------------- |  |
| 102 | የሚኖሩበት አካባቢ | ገጠር |  |
|  |  | ከተማ |  |
| 103 | የምን ሀይማኖቶ ተከታይ ነዎት? | ኦርቶዶክስ ክርስቲያን |  |
|  |  | ሙስሊም |  |
|  |  | ፕሮቲስታን |  |
|  |  | ካቶሊክ |  |
|  |  | ሌላ ካለ (ይገለጽ)------- |  |
| 104 | የትዳር ሁኔታ? | ያላገቡ |  |
|  |  | ያገቡ |  |
|  |  | የፈቱ |  |
|  |  | የትዳር አጋር በሞት የተለየ |  |
| 105 | የትምህርት ድረጃወት? | ያልተማርች |  |
|  |  | 1ኛ ደረጃ |  |
|  |  | 2ኛ ደረጃ |  |
|  |  | ዲፕሎማ |  |
|  |  | ዲግሪ እና ከዝያ በላይ |  |
| 106 | የእርስዎ ስራ ምንድን ነዉ | ነጋዴ |  |
|  |  | የመንግስት ሰራተኛ |  |
|  |  | የቤት እመቤት |  |
|  |  | የግል ሰራተኛ/ተከጣሪ |  |
|  |  | ተማር |  |
|  |  | ሌላ ካለ ----------- |  |

**ክፍል2፡ በአዊ ዞን በተመረጡ የመንግስት ጤና ተቋማት ለሚገኙ ኤች አይቪ ቫይረስ በደማቸዉ ለሚገኝባቸዉ ነፍሰጡር እና የሚያጠቡ እናቶች የኤች ኤይቪ ኤድስ ቫይረስ በደም ዉስጥ መኖሩን ለሌላ ሰዉ ማሳወቅን የሚዳስስ መጠይቅ**

| ተ.ቁ | ጥያቄ | መልስ | ዝለል/ሂድ |
| --- | --- | --- | --- |
| 201 | የኤች ኤይቪ ኤድስ ዉጤትሽን ለሌላ ሰዉ አሳዉቀሻል? | አወ የለም | የለም ካሉ  ቁ.304 |
| 202 | ለማን ነዉ ያሳወቅሽ? | ለትዳር አጋር  ለጓደኛ ,ለቤተሰብ ሌላ-------- |  |
| 203 | መድሃኒቱን በትክክል እና በስዓቱ እንድትወስጂ  ያግዙሻል? | አወ አይደለም |  |
| 204 | የትዳር አጋርሽ /ባለቤትሽ ኤች ኤይቪ ኤድስ በደሙ  ዉስጥ አለበት? | አለበት  የለበትም  አላዉቅም | የለበትም  /አላዉቅም  ወደተ.ቁ  306ይሂዱ |
| 205 | የፀረ ኤች ኤይቪ ኤድስ መድሃኒት እየወሰደ ነዉ? | አወ . የለም አላዉቅም |  |

**ክፍል 3፤ በአዊ ዞን በተመረጡ የመንግስት ጤና ተቋማት ለሚገኙ ኤች አይቪ ቫይረስ በደማቸዉ ለሚገኝባቸዉ ነፍሰ ጡር እና የሚያጠቡ እናቶች ከኤች ኤይቪ ኤድስ በሽታ እና ከህክምናዉ ጋር የተያያዙ መጠይቆ ች**

| ተ.ቁ | መጠይቅ | ምር ጫ | ዝለል/ሂድ |
| --- | --- | --- | --- |
| 301 | የኤች ኤይቪ ኤድስ መድሃኒት መዉሰድ ሲጀምሩ የአለም የጤና  ድርጅት ባወጣዉ የበሽታዉ ደረጃ የትኛዉ ላይ ነበሩ? | ደረጃ 1  ደረጃ 2  ደረጃ 3  ደረጃ 4 | እባከዎን  የታካሚዋን  ካርድ ካርድ  ይመልከቱ |
| 302 | በአሁኑ ስዓት የአለም የጤና ድርጅት ባወጣዉ የኤች ኤይቪ  ኤድስ በሽታ ደረጃ የትኛዉ ላይ ይገኛሉ? | ደረጃ 1  ደረጃ 2 | በመረጃ  ሰብሳቢዉ  የሚሞላ |
| 303 | የሲዲፎር /CD4/ መጠን ተለክቶ ወይም የኤች ኤይ ቪ ኤድስ  የበሽታ ደረጃ መሰረት ተደርጎ ነዉ መድሃኒቱን የጀመርሽዉ? | አወ  አይደለም |  |
| 304 | መድሃኒቱን ሲጀምሩ የነበረዎት የሲዲፎር /CD4/መጠን  ስንት ነበር? | --------- | ካርዱን  ይመልከቱ |
| 305 | ያለ ሲዲፎር መጠን ወይም የበሽታዉ ደረጃ የፀረ ኤች ኤይቪ ኤድስ መድሃኒት ማስጀመር በሚባለዉ ምርጫላይ አንቺ መዉሰድ  የጀመርሽ ዉመቼ ነ ዉ? | በእርግዝና ጊኤ  በጡት ማትባት ጊዜ  ከርግዝና በፊት |  |
| 306 | ኤች ኤይ ቪ ኤድስ እንደተመረመሩ ቫይረሱ ለተገኘባቸዉ ነፍሰ  ጡር እና ለሚያጠቡ እናቶች የህይወት ዘመን የፀረ ኤች  ኤይ ቪ ኤድስ መድሃኒት ማስጀመር በሚባለዉ ምርጫ ላይ  ያጋጠመሽ ፈታኝ ችግር ነበር ? | አወ  አይደለም | የለም ከሆነ ወደ  ተ.ቁ 308  ይሂ ዱ |
| 307 | ፈታኝ ነገሮች ምንድን ናቸዉ | -ወዲያዉኑ መድሃኒቱን  ለመጀመር /ለመወሰን መቸገር  -የመድሃኒቱን የጎንዮሽ ጉዳት  -እገለላለሁ የሚል ስጋ ት  -ለትዳር አ ጋር /ጓደኛ  ለመናገር መቸገ ር  ሌላ ----------- |  |
| 308 | ኤች ኤይ ቪ ኤድስ ቫይረሱ በደምሽ ዉስጥ መገኘቱን  ያወቅሽዉ መቼ ነበር ? | -ካሁን በፊት ታዉቅ ነበር  -በዚህ እርግዝና ግዜ የተገኘ  -ጡት በምታጠባበት ግዜ |  |
| 309 | የፀረ ኤች ኤይ ቪ ኤድስ መድሃኒቱ እንዴት እንደሚወሰድ  የምክር አገልግሎት አግኝተሽ ነበር ? | አወ  አላገኘሁም |  |
| 310 | የ መድሃኒቱ የጎንዮሽ ጉዳት አጋጥሞሽ ያዉቃል? | አወ  የለም | የለም ከሆነ ወደ  401 ይሂ ዱ |
| 311 | ምንድን ነዉ ያጋጠመሽ የጎንዮሽ ጉዳት? | -ማቅለሽለሽ  -ማስታወክ  -እራስ ማዞር  -የቆዳ ማሳከክ  -የእጅ እና የእግር መዳፍ  ቀለም መቀየር  ሌላ ------- |  |
| 312 | የመድሃኒቱ የጎንዮሽ ጉዳት ካጋጠመሽ በኋላ  መድሃኒቱ ተቀይሮልሻ ል? | አወ  የለም |  |

ክፍል 4፡ የትዳር አጋር ተሳትፎ ከእናት ወደ ልጅ ኤች አይ ቪ ቫይረስ እንዳይተላለፍ በሚደረገዉ ህክምና በአዊይ ዞን በተመረጡ የመንግስት ጤና ተቋማት ለሚገኙ ኤች አይቪ ቫይረስ በደማቸዉ ላለባቸዉ ነፍሰ ጡር እና የሚያጠቡ እናቶች መጠይቅ

| ተ.ቁ | የ ትዳር አጋርተሳ ትፎጥያቄዎች | አዎ | አይደለም |
| --- | --- | --- | --- |
| 401 | በቤት ዉስጥ ጉዳዮች ላይ የትዳር አጋርዎ ጋር ዉሳኔ ይጋራል |  |  |
| 402 | ከትዳር አጋሩ/ባለቤትዎ ጋር በግብረስጋ ግንኙነት ግዜ ስለ ከንዶም አጠቃቀም ዉይይት  ያደረጋሉ |  |  |
| 403 | ከእናት ወደ ልጅ ኤች አይቪ ቫይረስ እንዳይተላለፍ የፀረ ኤች ኤይቪ ኤድስ መድሃኒት በቀን ዉስጥ ስንት ጊዜ እንደሚወሰድ ያዉቃል |  |  |
| 404 | የፀረ ኤች ኤይቪ ኤድስ መድሃኒት ለመዉሰድ ከባለቤትዎ ጋር ጤና ተቋም አብሮ ይመጣል |  |  |
| 405 | ከእናት ወደ ልጅ ኤች አይቪ ቫይረስ እንዳይተላለፍ የሚያደረገዉን መድሃኒት  ስም ያዉቃል |  |  |
| 406 | ከእናት ወደ ልጅ ኤች አይቪ ቫይረስ እንዳይተላለፍ የሚያደረገዉን መድሃኒት  መጠን /dose/ያ ዉቃል |  |  |
| 407 | ስለቅ ድመወሊድ ወይም ድህረ ወሊድ ቀጠሮ ጥቅም ይወያያሉ |  |  |
| 408 | የትዳር አጋርዎን በቅድመ ወሊድ ወይም ድህረ ወሊድ ከእናት ወደ ልጅ ኤች አይቪ  ቫይረስ እንዳይተላለፍ ለሚደረገዉ ህክምና የገንዘብ ድጋፍ ያደርጋል |  |  |
| 409 | ከትዳር አጋጋር ከእናት ወደ ልጅ ኤች አይቪ ቫይረስ እንዳይተላለፍ በሚደረገዉ  ህክምና ይከታተላል |  |  |
| 410 | የትዳር አጋሩን /ባለቤቱን የቅድመ ወሊድ/ድህረ ወሊድ ክትትል እንድታደርግ  ያስታዉሳል |  |  |

ክፍል 5: የእናቶችን እውቀት የሚመዝን ቃለ መጠይቅ / የኤች ኤይቪ ኤድስ ቫይረስ በደማቸዉ ዉስጥ ለሚገኝባቸዉ ነፍሰ ጡር እና የሚያጠቡ እናቶች ያለሲዲፎር መጠን የህይወት ዘመን የፀረኤች ኤይቪ ኤድስ መድሃኒት ማስጀመር በሚባለዉ የህክምና ምርጫ ጋር የተያያዘ ጥያቄዎች

| ተ.ቁ | ጥያቄየዎች | መልስ | ሂድ/ዝልለ |
| --- | --- | --- | --- |
| 501 | የኤች አይቪ ኤድስ መተላለፍያ መንገዶችን ያውቃሉ? | አዎ  አላዉቅም |  |
| 502 | ኤች አይቪ ቫይረስ ከእናት ወደ ልጅ እንዳይተላለፍ መከላከያ አለው ብለው ያስባሉ? | አዎ  የለዉም |  |
| 503 | ስለ ኤች አይቪ ቫይረስ በደማቸዉ ዉስጥ ለሚገኝባቸዉነፍሰ ጡር እናቶች ያለምንም  ቅድመ ሁኔታ የህይወት ዘመን የፀረ ኤች ኤይቪ ኤድስ መድሃኒት መጀመር  እንዳለበት ሰምተዉያ ዉቃሉ? | አዎ  አላቅም |  |
| 504 | ኮንዶም በአግባቡ እና በትክክል ከተጠቀሙ ኤች ኤይቪ ኤድስ ያለበትን የትዳር አጋርንም  ሊከላከል ይችላ ል? | አወ  አይደለም |  |
| 505 | የፀረ ኤች አይቪ መድሃኒት መዉሰድ ቫይረሱ ከእናትወደ ልጅ የመተላለፍ አቅሙን ይቀንሳል | አወ  አይደለም |  |
| 506 | የፀረ ኤች አይቪ መድሃኒት እያቆራረጡ መዉሰድ ከእናትወደ ልጅ እንዳይተላለፍ  ለሚደረገዉ ህክምና ዉጤታማነት ተፅዕኖ አለዉ | አወ  አይደለም |  |
| 507 | የፀረ -ኤች ኤይቪ ኤድስ መድሃኒት በትክክል መዉሰድ ከኤይ አይቪ ኤድስ ጋር ከተያያዙ  ተጓዳኝ በሽታዎቸ መከላከል ይቻላል? | አወ  አይደለም |  |
| 508 | የትዳር አጋር /ጓደኛ ድጋፍ መኖር የፀረ ኤች ኤይቪ ኤድስ መድሃኒቱን በቁርጠኝት እና  በእምነት ለመዉሰድ ምንም አስተዋፅኦ የለዉም | አወ  አይደለም |  |

**ክፍል 6፡ ለፀረ ኤች ኤይቪ ኤድስ መድሃኒት ጥብቅ እምነት እና ቁርጠኝት መኖርን የሚመለከቱ ጥያቄዎች፡፡ በአዊ ዞን በተመረጡ የመንግስት ጤና ተቋማት ለሚገኙ ኤች አይቪ ቫይረስ በደማቸዉ ለሚገኝባቸዉ ነፍሰጡር እና የሚያጠቡ እናቶች የተዘጋጀ**

**የጥናቱ ተሳታፊ ግላዊ ገለፃ**

| ተ.ቁ | መጠይቆ ች | መልስ |
| --- | --- | --- |
| 601 | የፀረ ኤች ኤይቪ ኤድስ መድሃኒትሽን ስትወስጂ አንዳንድ ግዜ ለማስታወስ ተቸግረሽ  ነበር ? | አወ  አይደለም |
| 602 | ጥሩ ስሜት በሚሰማሽ ወቅት አንዳንድ ግዜ መድሃኒትሽን አቋርጠሸ ታዉቂያለሽ | አወ  አይደለም |
| 603 | ብዙ ታካሚዎች የፀረ ኤች ኤይቪ ኤድስ መድሃኒት እንደታዘዘላቸዉ ለመዉሰድ ሲቸገሩ  ይታያ ል:: ባለፉት ሶስት ቀናት ዉስጥ መዉሰድ ያለብሽን መድሃኒት አቋርጠሸ ነበር ? | አወ  አይደለም |
| 604 | አንዳንድ ግዜ መድሃኒቱን በምትወስጂበት ወቅት የባሰ ችግር ቢያጋጥምሽ ፤  መደሃኒቱን መዉሰድሽን ታቋርጫለሽ ? | አወ  አይደለም |

**ሴዛ V፣ አዊጚሾ ኪስስትኹ (ኬዬርስትኹ) ስምምነቱ ስታ ንባሩ ቅፅ**

አዊ ዞንዳ ሜሬትስቱንኩ መንግስትኩ ቲኑ ታኮምዳ አግስታንኩ ኤች አይቪ ኤዲስ ብሪዳ ዝኩንኩ ሼርካ ስታ፣ ፃኹፃንትካ ቹትካስ አዜጌጅስትኹ 2012ም.ዓ:: ጌርክ___ካሲው መላይ ቴርቼፍ(ኩትር)____________ቲኑ ተኮሙ ስም___________________________

**ንባሩ ጊሊፂ ቅፅ ስታ ስምምነት:**

ስምኪ __________ስቴ ። ባህር ዳር ዩንቨርስቲዳ ህክምኒው ፈካሊቲ ካሜንፅጙ ትምርት ቤንዳ ያኽስታውሳ ምርምሬ ንጝኪ ኤች አይቪ ኤዲስ ቾዴስ ጄርሾ ካይጛቲታ ያኽስታው ህክምኒዳ ኤች አይቪ ኤዲስ ብሪዳ አግስታንኩ ሼርካ ቹትካ ዳማኪ ፍንቲኒ(ቅድማ) ሁኔታ ጋታ (ሲዲ ፎ ር ሜቴን ስታ ቑንዚው ደረጂ) ህይዎቱሳ ዘመኖ(አሜቶ) ካፃኑ ኤች አይቪ ኤዲሱሳ እጆ ጄሜርፅጝ ነው ምርቺዳ ቹትካ ዝኩኹሳ ኩርፄንቶ ስታ ካፅጙሳ ድምክኒሱላ እሊኩ ምትጙንኩ ዲብካዳ ያኽስታው ንባሩ ሰበሰብጜኽ። እንዳ ምርምሪዳ አሴቴፍጛላ ኒኒኪ ዳማኪ አግልግሎቱዳ አግፅጝዳ ኬየርስታው (ሊሊት) ዲብ ዝኮላኪ። እይስታው እንክብካቢ ኬፄሌ። ይጋ ኑኒላ ሀሰቦ ኬየርጝስ ካሌና። እንቱ ጝሺ ምርምሪው ጝን ዌና ያኹኑውስ ባንታ ዳማኪ ስጋት(ጅፍንት) ዝኮቲታ።ዲብ ኩፂ ሌሜድስታየሱ ዲብ ጌቴሙኒጊ ነፃ ያኸከማ ጅፊስታኑሳ ካሴ ድኾንታ ፋኔ። እንዳ ምርምሪዳ አሴቴፍጝስ ዳማኪ ትክም እላ። ዲብ ኩፂ እን ምርምሪ አይኪ ጄር ኤች አይቪ ኤዲስስ እምስታማ ከሜንስትጝ እላኪ ነውሳ አለሜ ወኽትጝፅጝስ ኬፍ ንኹ ወይሚ ዝኮ።

**ምስቲሮ(ስርኩኔ) ማንዲጝ፣** ክዴስ ሳበሰብስታውሳ ንባሮ ምስቲርስ እምስታው ስታ ማረማርጛንቲዴስ እሊው አይኪ አቒ ካንታላኪ። አይኪ ኮዋ ካንተው ሰበሰብስትኹ ንባርዳ ስም ፃፊስታላኪ። ስሙውስ ባኒስ ሚስቲሩ ኮድ እይስታውያኽ። እሳ ኮዶ ማላዊ ማራመርጛንቲ ይኾቼኽ።አግስቱንኩሳ ዲብካዋ ጌሌፅጝስ፣ እንቱ (እንት) ምርምሪውሳ አግስቶ ማሊጝስ ፈቱኒጊ ምርምሪ ዊድስቱንዴስ ፈሌንጋ እንቱሊ(ክሊ) ኩስጝስ ካሊኔ።ድምክኒስኪላ ምርምሪውሳ ውቴቶ እሊኩሊ ምሁራንካሊ ስታ ሊሊትጙንኩ ማህበረሰብካሊ ኩስጝናው ያኼ።እሳ ፄውናው ምርምሪሳ ውቴቶ ማሊጝስ ፈያውኩስ ውላስ አቕስ ፁፍስ ስታ ሊሊትጙንኩ ድህረ-ጌፅስ ያኼ።

**ምርምሪኩ አሴቴፍጛንትካው አስሜምጝፂ ንባር**

ምርምሪ ካፀው ሰት ፤ እን ምርምሪ ፳፡፴ ዳኪካ ኺስቴ ካፄ። አጉዊዳ ጌሌፅስትኹሳ ንባሮ ክችክቺስ እንኮቓታ እርዳታ።ምርምሪውሳ ትክሞ፣ ታምፃውሳ ጉዳቶ(ችግሮ)፣ ሚስቲሮ ማንዲጞ፣ ትክማ ትክሞ ስታ ካፀውሳ ሰቶ ካንተውስ እርዳጽታ። እሳ ምርምሬ ካንተውስ ዙሚትጝፅጝስ ወይኪ ካሲጝስ ፋታኑ ጉዳይ ዝኩኒጊ ዋኒ መረማርጛንቲው አድረሼ ሲፍናነ ጌሌፅኔ ስምኪ =ተገኘ ዋለ ስልኪ ኩትር፤ 0918581910 ኢ_ሜይል tegegnewale 10@gmail.com

**ምርምሪዳ አሴቴፍጝስ ፈካዴናማ? ሀ፣ ይጋ , ለ ፈታለኪ** ዙርሚ ይጋ ያኹኒጊ ኪፂል፤ አይ ፈታላኪ ያኹኒኪ ሜሴጌኒፅ ስታ ሲፋው ካሉ ካሲሾ ፌት። ንባሮ ሰበሰባንቲው ስም________________________________ ፊርሚ _________ ጌርክ **__________**

**ሴዛ VI ምርምሪው ካስካ**

**ቤን አምፕል(1): ማቢሪው አከባቢውስታ ኢኮኖሚው ካስካ።አዊ ዞን ሜሬትቱንኩ መንግስትኩ ቲኑ ታኮምዳ አግስታንኩ ኤች አይቪ ኤዲስ ብሪዳ ዝኩንኩ ሼርካ ስታ፣ ፃኹፃንትካ ቹትካስ አዜጌጅስትኹ**

| ቴር ቼፍ | ካስካ | ዙርሚ | ፌት/ ካ |
| --- | --- | --- | --- |
| 101. | እድሚ ውኻይ? | _______________________ |  |
| 102. | ዝኪኑ አከባቢ ወዳይ ? | ጌፄር፣  ኬቴም |  |
| 103. | እንዳር አይማኖቶይ ሲፍጝታው? | ኦርቶዶክስ፣ እስሊምኒ፣ ፕሮቴስታንት፣ ካቶሊክ፣ እሊው ዝኩኒጊ _______ |  |
| 104. | ትዳሩ ኹኔቲሳ? | ሚፂያስኻ፣ ሚፄኪኾ፣ ትፍታስኪኾ፣ ጝንዌና ክራስኩኻ |  |
| 105. | ትምርቱ ደረጂ? | ክንቲያስኻ፣ እምፕላንቲ ደረጂ፣ ላጛንቲ ደረጂ፣ ቴክኒክና ሙያ፣ ዲፕሎማ፣ ዲግሪስታ አንዴስ ጃላ_______ |  |
| 106. | ኩ እንፅኺ ዳማጚ ? | ጌፄና፣ መንግስቲ ስራቴና፣ ጝንቲ ፣ ግልቲ ስራቴና፣ ክንታንታ፣እሊው ዝኩኒጊ(ድቑይ)_______ |  |
| 107 | ምርምሪው አሴቴፍጛንታ ሼራማ፣ ፃኹፃንታይ? | _____________________ |  |

ቤን **ላጛ(2)**  አዊ ዞን ሜሬትስቱንኩ መንግስትኩ ቲኑ ታኮምዳ አግስታንኩ ኤች አይቪ ኤዲስ ብሪዳ ዝኩንኩ ሼርካ ስታ፣ ፃኹፃንትካ ቹትካ ኤች አይቪ ኤዲስ ብሪዳ ዝኩንኩ አኽጞ እሊኩስ አቕስ ድኹጞ ጌሌፃንኩ ካስካ

| ቴር ቼፍ | ካስካ | ዙርሚ | ፌት/ ካ |
| --- | --- | --- | --- |
| 201, | ኤች አይቪ ኤዲሱሳ ውቴቶ እሊኩስ አቕስ ድኹታማ? | ይጋ፣  እላኪ |  |
| 202. | አይሲ ድኹቱኺ? | ጝን ዌና ጝርጂስ ፣ ጉዲኒስ፣ ቤተሰብስ(ጝንኩ አቕስ )፣ እሊኩስ_________ |  |
| 203. | እጆ ክችክቺስ ስታ ሰትስ ካፄታ እርደታነማ? | ይጋ፣  እላኪ |  |
| 204. | ጝርጂዳ (ኬራዳ) ኤች አይቪ ኤዲስ ዝኩኹማ? | ይጋ ዝኮ፣ እላኪ |  |
| 205. | ኤች አይቪ ኤዲሱሳ እጆ ካፀማጊማ | ይጋ ፣ እላኪ |  |

ሹኻ(3). አዊ ዞን ሜሬትስቱንኩ መንግስትኩ ቲኑ ታኮምዳ አግስታንኩ ኤች አይቪ ኤዲስ ብሪዳ ዝኩንኩ ሼርካ ስታ፣ ፃኹፃንትካ ቹትካ ኤች አይቪ ኤዲስ ቑንዚ ስታ እክምኒሊ ምትጙንኩ ካስካ

| ቴር ቼፍ | ካስካ | ዙርሚ | ፌት/ ካ |
| --- | --- | --- | --- |
| 301. | ኤች አይቪ ኤዲሱሳ እጆ ካፅጞ ጄሜርቱስ አሌሙ ቲኑ ድርጅት ፍሽኹስ ቑንዚውስ ደረጂስ ወዳይ እሽቲኹ | ደረጂ 1 ደረጂ 2  ደረጂ 3 ደረጂ 4 | ቲኬምስታንቲውሳ ካርዶ ከንት |
| 302. | ጝሺሱውስ ሰዓትዳ አሌሙ ቲኑ ድርጅት ፍሽኹስ ቑንዚውስ ደረጂስ ወሺኒዳ ደረጂዳ አግስቴ? | ደረጂ 1  ደረጂ 2 | ንባሮ ሰብሰባንቲስ ወኽስታው |
| 303. | ሲዲ ፎር ሜቴን ከንፅስታማም ወይኪ በሺቲው ደረጂስማ እጆ ጄሜርትኹይ? | ይጋ፣  ጋቲዊ |  |
| 304. | እጆ ጄሜርቱስ ሲዲ ፎር ሜቴን ውኻይ እሺኹ? | _____________ | ካርዶ ካንት |
| 305. | ሲዲ ፎር ሜቴን ጋታ ወይኪ በሺቲው ደረጂ ጋታ ኤች አይቪ ኤዲሱሳ እጆ ጄሜርፅጝ ዝ ኮ ነው ምርቺስ እንት ካፅጞ ጄሜርትኹ ወኒ | ሼራ ዝኬኪስ፣ አንጎ ፃኹፂስ፣ ሼራ አኽጝዴስ ፍናዋ |  |
| 306. | ኤች አይቪ ኤዲሶ መረመርስትካማ ቫይረስ ዝኩክስ ሼራካስታ፣ አንጎ ፃኹፃንትካ ቹትካስ ሂወቱሳ አሜቶ ኤች አይቪ ኤዲሱሳ እጆ ጄሜርፅጝ ዝኮ ነው ምርቺዳ ጌቴምኹ ችግር ዝኮማ? | ይጋ፣  እላኪ | እላኪ አኹኒጊ ዙርሚ ቴር ቼፍ 308 ካሳን |
| 307. | ጌቴሙንኩ ችግርካ እንዳርካ እንዳርካይ፤ | እሜስታ እሜ እጆ ጄሜርጝስ ስታ ወሴንጝስ ቼጌርስትጝ ፣  እጁ ጎኒዮሽ ጉዳት፣  ኮውሳ ፄጌራማ ጅፊስትጝ፣  ጝርጂስ አኹኪ ጉዲኒስ ድኹጝስ ቼጌርስትጝ ፣ እሊው _____ |  |
| 308. | ኤች አይቪ ኤዲስ ቫይረስ ክዳ ዝኩጞ  ማሊኹ ዋኒ? | እንዲስ ፍናዋ ማላ፣ እንጚዳ ሼርትዳ አግስቱኽ፣ አንጎ ፃኹፂስ |  |
| 309. | ኤች አይቪ ኤዲሱ እጆ ወታፄውታታ ካፄው አኽጝ ኩስጚው አግልግሎት እይስታሺኾማ፤ | ይጋ፣  እላኪ እይስታያለኪ |  |
| 310 | እጁ ጎኒዮሽ ጉዳት ጌቴማ አቑኻማ እንዴስ ፍና? | ይጋ፣  እላኪ | እላኪ አኹኒጊ ዙርሚ ቴር ቼፍ 501 ካሳን |
| 311 | ዳማጚ ታምብኹ እጁ ጎኒዮሽ ጉዳት? | ላኽላኽፅጝ፣ልቒፅጝ፣ጛሬ ዙራዙርፅጝ፣ አከላቶ ሳስፅጝ፣ ታፉስታ ልኩ ክቻ ኬሌም ኬየርጝ ፣ እሊው ዝኩኒጊ____ |  |
| 312 | እጁ ጎኒዮሽ ጉዳት ጌቴሙንዴስ ፈሌንጋ እጁ ኬየርስታይኾማ? | ይጋ፣ እላኪ |  |

**ቤን** 4 **አጋሩሳ ተሳትፎ ቾዴስ ጄርሾ ኤች አይቪ ኤዲስ ፌያፌይጛቲታ አኽስታውስ ህክምኒስ አዊ ዞንዳ ሜሬጽቱንኩ መንግስትኩ ቲኑ ታኮምዳ አግስታንኩ ኤች አይቪ ኤዲስ ብሪዳ ዝኩንኩ ሼርካ ስታ፣ ፃኹፃንትካ ቹትካስ አዜጌጅስትኹ**

| ቴር ቼፍ | ትዳሩ አጋሩ(ጝርጂው) ተሳትፉ ካስካ | ይጋ(አዎ) | እላኪ(ጋቲዊ) |
| --- | --- | --- | --- |
| 401. | ጝናኻስኩዳ ጉዳይካዳ ኹናሊ ውሳኔ ወሴንታናማ |  |  |
| 402. | ጝን ዌና ጝርጂሊ አረኹንቶ ፄውታኑዳ ሰትዳ ኮንደሙሳ አተካከሞ ኩስጝትካ ታቓካማ |  |  |
| 403. | ኤች አይቪ ኤዲስ ቾዴስ ጄርሾ ካይጛቲታ ኤች አይቪ ኤዲስ እጁ ጌርክስ ውኺኒ ስጙስታው አኽጞ ማለካማ |  |  |
| 404. | ኤች አይቪ ኤዲስ እጁ ካፅጝስ ጝንቲ ኹናሊ አብራ ቲኑሳ ተኮሞ ቲንቴማ |  |  |
| 405. | ቾዴስ ጄርሾ ካይጛቲታ ካፅስታውሳ ኤች አይቪ ኤዲስ እጁሳ ስሞ ማሌማ፣ |  |  |
| 406. | ቾዴስ ጄርሾ ካይጛቲታ ካፅስታውሳ ኤች አይቪ ኤዲስ እጁሳ መተኖ (ዶዝ) ማሌማ |  |  |
| 407. | ክምንትዴስ ፍናዋ አኹኪ ፈሌጋ ዝኩኹሳ ቀጠሩሳ ትክሞ ኩስጝታናማ |  |  |
| 408. | ጝንሳ ኹናዋ ክምንትዴስ ፍናዋ አኹኪ ፈሌጋ ኤች አይቪ ኤዲስ ቾዴስ ጄርሾ ካይጛቲታ ያኽስታውስ ህክምኒስ ጌንዘቡሳ እርዳቴ ፄውቴማ |  |  |
| 409. | ኤች አይቪ ኤዲስ ቫይረስ ቾዴስ ጄርሾ ካይጛቲታ ያኽስታው ሳ ህክምኔ አብራ ሲፍጝቴማ |  |  |
| 410. | ኹዋ ክምንትዴስ ፍናዋ አኹኪ ፈሌጋ ሲፍጜ ፄውታታ አስተወስፂማ |  |  |

ቤን **:አንኮ (5** )ቹትካውሳ እውከቶ ሚንዛነው ካሲ፤ኤች አይቪ ኤዲስ ብሪዳ አግስታንኩ ሼርካ ቹትካ ዳማኪ ፍንቲኒ(ቅድማ) ሁኔታ ጋታ (ሲዲ ፎ ር ሜቴን ስታ ቑንዚው ደረጂ) ህይዎቱሳ ዘመኖ(አሜቶ) ካፃኑ ኤች አይቪ ኤዲሱሳ እጆ ጄሜርፅጝ ነው ምርቺሊ ምትጙንኩ ካስካ::

| ቴር ቼፍ | ካስካ | ዙርሚ | ፌት/ ካ |
| --- | --- | --- | --- |
| 501. | ኤች አይቪ ኤዲሱሳ ፌያፌይጝፄ ዳዶ ማሌማ | ይጋ (አዎ)፣ እላ ማለያለኪ |  |
| 602 | ኤች አይቪቫይረ ዝኩት ሼራ ኹና ቾዴስ ጄርሾ ቫይረሶ ፌያፌይጝፄውሳ ፄጌሬማ? | ይጋ ፣ ፌያፌይጝፃላ |  |
| 503 | ኤች አይቪ ኤዲስ ቾዴስ ጄርሾፌያፌይጛቲታ ካለካልጝፂ ዳድ ዝኮ ኔታ አሴብቴማ? | ይጋ,  እላኪ |  |
| 504 | ኮንዶሞ አግበብስ ስታ ክችክቺስ ቴኬምስትጝ ኤች አይቪ ኤዲስ ዝኩክሳ አረኹናዋ ከለካልጝስ ካሌ? | ይጋ,  እላኪ |  |
| 505 | ች አይቪ ኤዲሱሳ እጆ ከፅጝ ቫይረስ ቾዴስ ጄርሾ ፌያፌይጛውሳ ሜቴኖ(አክሞ) ኬኔሴ? | ይጋ,  እላኪ |  |
| 506 | ኤች አይቪ ኤዲሱሳ እጆ አኮሬትጝፃማ ከፅጝ ቫይረስ ቾዴስ ጄርሾ ፌያፌይጛቲታ ፄውስታውደ ውቴታመነትደ ተፅኑ ዝኮ? | ይጋ,  እላኪ |  |
| 507. | ኤች አይቪ ኤዲሱሳ እጆ ክችክቺስ ቴኬምስትጝ(ስጙጝ) ኤች አይቪ ኤዲስሊ ምትጙንኩ ቑንዝካዴስ ኦፖርጩኒስቲክ ኢንፈክሽን ከለካልጝስ ካሊስቴ? | ይጋ,  እላኪ |  |
| 508 | አረኹናው ወይኪ ጉዴንካው እርዳቲ ዝኩጝ ኤች አይቪ ኤዲሱሳ እጆ ክችክቺስ ኩርፄንት ስ ስታ እምነት ስ ስጙጝስ አኹኪ ካፅጝስ ዳማኪ አስተወፁ እላኪ? | ይጋ,  እላኪ |  |

**ቤን ላጜታ (6)** ኤ**ች አይቪ ኤዲሱ እጁዳ ዝኩኹሳ እምኔቶስታ ኩርታኝኔቶ ካንፅፃንኩ ካስካ።አዊ ዞን ሜሬትቱንኩ መንግስትኩ ቲኑ ታኮምዳ አግስታንኩ ኤች አይቪ ኤዲስ ብሪዳ ዝኩንኩ ሼርካ ስታ፣ ፃኹፃንትካስ አዜጌጅስትኹ**

| ቴር ቼፍ | ካስካ | ዙርሚ |
| --- | --- | --- |
| 601. | ኤች አይቪ ኤዲሱሳ እጆ ስጙቱስ እምፕላቺ እምፕላቺ አስቴዌስፅጝስ ቺጊርስቴ ታቓማ | ይጋ፣ (ጋቲዊ)ቺጊርስታ አቔያላ |
| 602. | ሼጊ ስሜት እንኮኽስቱኽዳ ወክትዳ እምፕላኒ እምፕላኒ እጆ አ ኮሬትጝፄ ታቓማ | ይጋ፣ እላኪ |
| 603. | ሜንችካ አቕ ኤች አይቪ ኤዲሱሳ አዜዝስቱኑው እጆ ካፅጝስ ቼጌርስታኒስ ካንቴና። ፌይኹዳ ሹኻ ጌርካኻዳ ካፄው እሽቲኹሳ እጆ አኮሬትጝፄማ | ይጋ (ዋ?)፣ እላኪ |
| 604. | እምፕላኒ እምፕላኒ እጆ ካፄውዳ ሰትዳ ባስኹ ችግር  ጌቴሙኒጊ አ ኮሬትጝፂማ | ይጋ፣ እላኪ |
